# Efficacy and safety of rozanolixizumab in patients with muscle-specific tyrosine kinase autoantibody-positive generalised myasthenia gravis: a subgroup analysis of the randomised, double-blind, placebo-controlled, adaptive phase III MycarinG study

**DOI:** 10.1177/17562864241273036

**Published:** 2024-09-12

**Authors:** Ali A. Habib, Sabrina Sacconi, Giovanni Antonini, Elena Cortés-Vicente, Julian Grosskreutz, Zabeen K. Mahuwala, Renato Mantegazza, Robert M. Pascuzzi, Kimiaki Utsugisawa, John Vissing, Tuan Vu, Heinz Wiendl, Marion Boehnlein, Bernhard Greve, Franz Woltering, Vera Bril

**Affiliations:** MDA ALS and Neuromuscular Center, University of California, 200 South Manchester Avenue, Suite 110, Irvine, Orange, CA 92868, USA; Peripheral Nervous System & Muscle Department, Pasteur 2 Hospital, Centre Hospitalier Universitaire de Nice, Université Côte d’Azur, Nice, France; Department of Neuroscience, Mental Health and Sensory Organs (NESMOS), Sapienza University of Rome, Rome, Italy; Neuromuscular Diseases Unit, Hospital de la Santa Creu i Sant Pau, Barcelona, Spain; Precision Neurology of Neuromuscular Diseases, Department of Neurology, University of Lübeck, Lübeck, Germany; Department of Neuromuscular Medicine, Epilepsy and Clinical Neurophysiology, University of Kentucky, Lexington, KY, USA; Department of Neuroimmunology and Neuromuscular Diseases, Fondazione IRCCS, Istituto Nazionale Neurologico Carlo Besta, Milan, Italy; Department of Neurology, Indiana University School of Medicine, Indiana University Health, Indianapolis, IN, USA; Department of Neurology, Hanamaki General Hospital, Hanamaki, Japan; Department of Neurology, Copenhagen Neuromuscular Center, Rigshospitalet, University of Copenhagen, Copenhagen, Denmark; Department of Neurology, University of South Florida Morsani College of Medicine, Tampa, FL, USA; Department of Neurology, Institute of Translational Neurology, University Hospital Münster, Münster, Germany; UCB Pharma, Monheim am Rhein, Germany; UCB Pharma, Monheim am Rhein, Germany; UCB Pharma, Monheim am Rhein, Germany; Department of Neurology, University Health Network, Toronto, ON, Canada

**Keywords:** Muscle-specific tyrosine kinase, muscle-specific tyrosine kinase autoantibody positive, myasthenia gravis, rozanolixizumab

## Abstract

**Background:**

Muscle-specific tyrosine kinase (MuSK) autoantibody-positive (Ab+) generalised myasthenia gravis (gMG) is a rare and frequently severe subtype of gMG.

**Objectives:**

To assess the efficacy and safety of rozanolixizumab in the subgroup of patients with MuSK Ab+ gMG in the MycarinG study.

**Design:**

A randomised, double-blind, placebo-controlled phase III study.

**Methods:**

Patients with acetylcholine receptor (AChR) Ab+ or MuSK Ab+ gMG (aged ⩾18 years, Myasthenia Gravis Foundation of America Disease Class II–IVa, Myasthenia Gravis Activities of Daily Living [MG-‍ADL] score ⩾3.0 [non-ocular symptoms], Quantitative Myasthenia Gravis score ⩾11.0) were randomly assigned (1:1:1) to receive once-weekly subcutaneous infusions of rozanolixizumab 7 mg/kg, rozanolixizumab 10 mg/kg or placebo for 6 weeks, followed by an 8-week observation period. Randomisation was stratified by AChR and MuSK autoantibody status. The primary study endpoint was change from baseline to Day 43 in MG-ADL score. Treatment-emergent adverse events (TEAEs) were also assessed.

**Results:**

Overall, 200 patients were randomised, of whom 21 had MuSK Ab+ gMG and received rozanolixizumab 7 mg/kg (*n* = 5), 10 mg/kg (*n* = 8) or placebo (*n* = 8). In patients with MuSK Ab+ gMG, reductions from baseline to Day 43 in MG-ADL scores were observed: rozanolixizumab 7 mg/kg least squares mean (LSM) change (standard error), –7.28 (1.94); 10 mg/kg, –4.16 (1.78); and placebo, 2.28 (1.95). Rozanolixizumab 7 mg/kg LSM difference from placebo was −9.56 (97.5% confidence interval: −15.25, −3.87); 10 mg/kg, −6.45 (−11.03, –1.86). TEAEs were experienced by four (80.0%), five (62.5%) and three (37.5%) patients with MuSK Ab+ gMG receiving rozanolixizumab 7 mg/kg, 10 mg/kg and placebo, respectively. No patients experienced serious TEAEs. No deaths occurred.

**Conclusion:**

This subgroup analysis of adult patients with MuSK Ab+ gMG enrolled in the MycarinG study supports the use of rozanolixizumab as an effective treatment option for patients with gMG who have MuSK autoantibodies.

**Trial registration:**

ClinicalTrials.gov: NCT03971422 (https://clinicaltrials.gov/study/NCT03971422); EU Clinical Trials Register: EudraCT 2019-000968-18 (https://www.clinicaltrials‌register.eu/ctr-search/trial/2019-000968-18/GB).

## Plain language summary

Rozanolixizumab improved symptoms in people with anti-muscle-specific tyrosine kinase antibody-positive generalised myasthenia gravis in the MycarinG clinical study

Myasthenia gravis is a rare, chronic autoimmune disease affecting the communication between nerves and muscles. People with the disease experience fluctuating muscle weakness and fatigue, leading to problems with mobility, speaking, swallowing and breathing. The disease is called generalised when muscles other than those that move the eyes and eyelids are affected. It is caused by antibodies that attack a person’s own cells. Most people with the disease have antibodies against acetylcholine receptors (AChRs). However, some have antibodies against the muscle-specific tyrosine kinase (MuSK) protein and can experience more severe symptoms compared with people who have anti-AChR antibodies. Standard treatments for myasthenia gravis do not always work for people with anti-MuSK antibodies. The MycarinG study looked at whether rozanolixizumab was better than a placebo at treating the symptoms of adults with generalised myasthenia gravis and anti-AChR or anti-MuSK antibodies. Assessments measured disease severity and myasthenia gravis symptoms, such as physical fatigue, and how they affected daily activities. The study also looked at whether people receiving rozanolixizumab had any side effects. Here, we look at the group of people with anti-MuSK antibodies who took part in the MycarinG study. In total, 21 of the 200 people in the study had anti-MuSK antibodies. The symptoms of myasthenia gravis improved more in people with anti-MuSK antibodies who received rozanolixizumab than in those who received placebo. Common side effects with rozanolixizumab included headache, diarrhoea and feeling sick. No serious side effects were seen, and no patients died. The results show that rozanolixizumab is an effective treatment for people with generalised myasthenia gravis who have anti-MuSK antibodies. The results in this group of people are consistent with those seen in all people who took part in the study (with either antibody type).

## Introduction

Generalised myasthenia gravis (gMG) is a rare, chronic autoimmune disorder caused by impaired neurotransmission at the postsynaptic membrane of neuromuscular junctions (NMJ).^1–3^ The predominant manifestation is fluctuating and fatigable muscle weakness, which can be life-‍threatening if the respiratory or bulbar muscles are affected.^1,4,5^

While the majority of patients with myasthenia gravis (MG) have detectable antigen-specific autoantibodies directed against the acetylcholine receptor (AChR) on the postsynaptic membrane of the NMJ,^4,6^ a small proportion of patients with MG have muscle-specific tyrosine kinase (MuSK) autoantibodies (5%–8%).^4,7^ MuSK plays a central role in NMJ organisation and maintenance by facilitating AChR clustering.^1,4^ MuSK autoantibody-positive (Ab+) gMG is mainly immunoglobulin G4 (IgG4)-mediated; IgG4 autoantibodies prevent MuSK–low-density lipoprotein receptor-related protein 4 interaction, subsequently reducing AChR clustering at the NMJ, leading to impaired muscle contraction.^1,4,8^

The MuSK Ab+ gMG subtype exhibits a strong female predominance, and a higher overall prevalence is observed in Southern Europe compared with Northern Europe. In contrast to AChR Ab+ gMG, disease onset is early, with a peak incidence towards the end of the third decade.^7,9,10^

Patients with MuSK Ab+ gMG are considered to have a distinctive subtype of MG, which is frequently more severe than other subtypes.^7^ Onset of MuSK Ab+ gMG is usually acute, with rapid symptom progression within a few weeks, and typically affects the bulbar muscles.^7,9^ Due to the atypical onset and clinical features of the disease, including marked muscle atrophy, selective bulbar involvement and lack of symptom fluctuations, the diagnosis of MuSK Ab+ gMG can be challenging.^7,9,10^ These challenges further extend to the management of MuSK Ab+ gMG, with patients experiencing an often unsatisfactory response to some of the treatments typically used for AChR Ab+ gMG. For example, patients with MuSK Ab+ gMG show limited response to intravenous immunoglobulin (IVIg) and may experience worsening with acetylcholinesterase inhibitors.^6,7,9^ Consistent thymic abnormalities have not been reported in patients with MuSK Ab+ gMG; hence, thymectomy is not considered a therapeutic option.^6,11^ Furthermore, since immunoglobulin G (IgG) autoantibodies of the IgG4 subclass do not activate complement, the use of complement inhibitors is presumed ineffective in the MuSK Ab+ population.^6,9,12^ The limited treatment options available for patients with MuSK Ab+ gMG include plasma exchange therapy and rituximab, a CD20 inhibitor.^11,13^ Rituximab has been included in recommendations for the management of patients with MuSK Ab+ gMG whose response to initial immunotherapy is unsatisfactory, despite it not currently being licensed for this indication.^11,14^ Conventional immunosuppression therefore remains the cornerstone of treatment for MuSK Ab+ gMG.^7,9^

In recent years, progress has been made in the development of antigen-specific immunotherapies directed against the cells and immune pathways involved in MG pathogenesis.^4,6^ The reduction of IgG autoantibodies via inhibition of the neonatal Fc receptor (FcRn) is one such target for the treatment of MG.^15,16^ FcRn functions as a natural salvage and recycling mechanism that is responsible for prolonging the half-life of serum IgG molecules by preventing lysosomal IgG degradation.^16–19^ Inhibition of FcRn thus allows for the targeted reduction of IgG antibodies, including pathogenic autoantibodies of the IgG4 subclass implicated in MuSK Ab+ gMG pathogenesis.^19^

Rozanolixizumab is a humanised IgG4 monoclonal antibody that reversibly binds to FcRn with high affinity.^15,16^ In June 2023, rozanolixizumab was approved by the United States Food and Drug Administration for the treatment of adult patients with AChR Ab+ or MuSK Ab+ gMG.^20^ Rozanolixizumab has since been approved in Japan for the treatment of patients with gMG who inadequately respond to corticosteroids or non-corticosteroid immunosuppressants,^21^ and in Europe and the UK as an add-on to standard therapy for the treatment of gMG in adult patients who are AChR Ab+ or MuSK Ab+.^22,23^ The pivotal phase III MycarinG study (NCT03971422; EudraCT 2019-000968-18) established the efficacy and safety of rozanolixizumab in adults with AChR Ab+ or MuSK Ab+ gMG.^15^ Here, we report findings in the subgroup of patients with MuSK Ab+ gMG in the phase III MycarinG study.

## Materials and methods

### Study design and patients

MycarinG was a randomised, double-blind, placebo-controlled, parallel-group, two-stage adaptive phase III study in patients with AChR or MuSK Ab+ gMG; the full study design has been reported previously.^15^ In brief, patients were randomised 1:1:1 to receive subcutaneous infusions of rozanolixizumab 7 mg/kg, rozanolixizumab 10 mg/kg or placebo once a week for 6 weeks on top of their current gMG treatment (where permitted by the study inclusion criteria). Randomisation was stratified by the presence of AChR or MuSK autoantibodies. The 6-week treatment period was followed by an observation period of 8 weeks. Patients were then eligible to roll over into either of the open-label extension (OLE) studies: MG0004 (completed; NCT04124965; EudraCT 2019-000969-21) or MG0007 (completed; NCT04650854; EudraCT 2020-003230-20).^15^ Rescue therapy (IVIg or plasma exchange) was permitted at the investigator’s discretion for patients who experienced disease worsening. Patients who required and opted to receive rescue therapy during the treatment period stopped receiving the study drug and completed all remaining visits before moving into the observation period. If they subsequently required rescue therapy during the observation period, they were given the option to either enrol in an OLE study, providing at least 2 weeks had passed since receipt of rescue therapy, or receive rescue therapy and not be invited to enrol in an OLE study. Patients who completed the treatment period without receiving rescue therapy but required rescue therapy during the observation period could choose to enrol in an OLE study or receive rescue therapy and not be invited to enrol in an OLE study.

Full inclusion and exclusion criteria of the MycarinG study have been reported previously.^15^ Briefly, patients aged ⩾18 years with a diagnosis of gMG (Myasthenia Gravis Foundation of America Disease Class II–IVa), a Myasthenia Gravis Activities of Daily Living (MG-ADL) score ⩾3.0 (for non-ocular symptoms) and a Quantitative Myasthenia Gravis (QMG) score ⩾11.0 who had been considered by the investigator for additional therapy such as IVIg or plasma exchange were eligible for enrolment.

At screening, all patients were required to have a previously documented positive record of autoantibodies against MuSK or AChR from historical diagnostic tests. The presence of autoantibodies against MuSK or AChR was also assessed at study baseline using a clinical laboratory radioimmunoassay. Baseline assessment was for exploratory quantification and did not inform eligibility. MG-specific autoantibody status from the historical diagnostic tests was used to define MuSK autoantibody positivity for this subgroup analysis.

### Study endpoints

The primary efficacy endpoint was the change from baseline to Day 43 in MG-ADL score. Secondary efficacy endpoints included change from baseline to Day 43 in Myasthenia Gravis Composite (MGC), QMG and MG Symptoms Patient-Reported Outcomes (PRO) Muscle Weakness Fatigability, Physical Fatigue and Bulbar Muscle Weakness^24^ scores. MG-ADL response (based on the established clinically meaningful individual patient-level improvement from baseline of ⩾2.0 points)^25^ at Day 43 was also assessed. Other efficacy endpoints included MGC and QMG response (based on the clinically meaningful improvement of ⩾3.0 points)^26,27^ at Day 43, change from baseline in MG-ADL, MGC and QMG at each scheduled assessment during treatment and observation periods and minimal symptom expression (MSE; MG-ADL score of 0 or 1) during the treatment period.

Safety and tolerability outcomes included the occurrence of treatment-emergent adverse events (TEAEs) and TEAEs leading to study drug discontinuation. Pharmacodynamic outcomes were change from baseline in MG-specific autoantibodies, serum total IgG and IgG subclass concentrations. Other outcomes, including pharmacokinetic outcomes, have been described previously.^15^

### Statistical analysis

Full details of the statistical analyses conducted in the overall study population, including the calculation and justification of the sample size, have been described previously.^15^

MuSK Ab+ and AChR Ab+ gMG subgroup analyses were performed according to the randomly assigned treatment (randomised set). Evaluation of the primary and secondary efficacy endpoints in the subgroups was pre-specified and the endpoints were analysed using a mixed model for repeated measures, which included treatment group, baseline MG-‍ADL score, geographical region and treatment group by day as fixed factors, with study patient as a random effect. The model utilised an unstructured covariance pattern for the repeated measures. Based on the model, 97.5% confidence intervals (CIs) were reported. Safety analyses for the subgroups were carried out in the safety set, which included all randomly assigned patients who received at least one dose of rozanolixizumab, analysed according to the actual treatment received. All subgroup analyses and comparisons between the overall population and the subgroups were descriptive.

## Results

### Baseline demographics and characteristics

**Figure 1.**
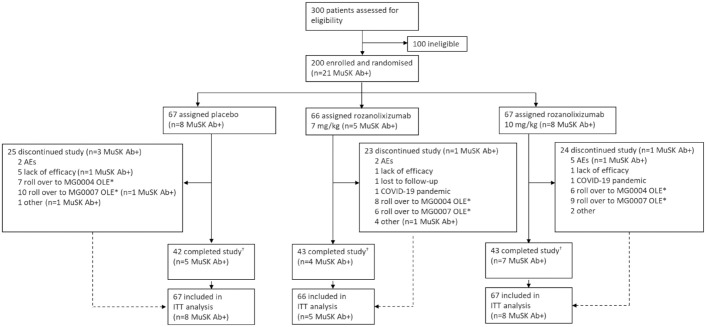
Trial profile and patient disposition. *Required rescue therapy (investigator judgement) during the observation period of MycarinG. ^†^Completed both the treatment and observation periods; 64 patients completed the treatment period in each of the placebo and rozanolixizumab 7 mg/kg groups and 62 patients completed the treatment period in the rozanolixizumab 10 mg/kg group. AE, adverse event; MuSK Ab+, muscle-specific tyrosine kinase autoantibody positive; ITT, intention-to-treat; OLE, open-label extension.

The MycarinG study took place over 29 months, with patients randomised between 3 June 2019 and 30 June 2021. In total, 200 patients received rozanolixizumab 7 mg/kg (66 [33.0%]), rozanolixizumab 10 mg/kg (67 [33.5%]) or placebo (67 [33.5%]; Figure 1). A total of 21 (10.5%) patients had a documented history of autoantibodies against MuSK (rozanolixizumab 7 mg/kg: *n* = 5 [7.6%]; rozanolixizumab 10 mg/kg: *n* = 8 [11.9%]; placebo: *n* = 8 [11.9%]). Of these 21 patients, six (28.6%) tested negative for autoantibodies against MuSK at baseline (rozanolixizumab 7 mg/kg: *n* = 1; rozanolixizumab 10 mg/kg: *n* = 4; placebo; *n* = 1). Two (9.5%) of the 21 patients also had a documented history of AChR autoantibodies (placebo: *n* = 1, MuSK Ab+/AChR Ab− at baseline; rozanolixizumab 10 mg/kg: *n* = 1, MuSK Ab−/AChR Ab+ at baseline).

All patients with MuSK Ab+ gMG treated with rozanolixizumab had Myasthenia Gravis Foundation of America Disease Class II or III at baseline (Table 1). Compared with the overall population, mean MG-ADL score at baseline was numerically greater in patients with MuSK Ab+ gMG. Within the MuSK Ab+ gMG subgroup, mean baseline MG-‍ADL score was numerically greater in those treated with rozanolixizumab 7 mg/kg compared with those receiving rozanolixizumab 10 mg/kg. A greater proportion of patients with MuSK Ab+ gMG were female and had experienced prior MG crisis, while a lower proportion had thymectomy compared with the overall population at baseline.

**Table 1.** Baseline demographic and clinical characteristics for patients with MuSK Ab+ gMG, AChR Ab+ gMG and the overall population (randomised set).

	MuSK Ab+ population*			AChR Ab+ population*			Overall population		
Category	Placebo (*n* = 8)^†^	RLZ 7 mg/kg (*n* = 5)	RLZ 10 mg/kg (*n* = 8)^†^	Placebo (*n* = 59)^†^	RLZ 7 mg/kg (*n* = 60)	RLZ 10 mg/kg (*n* = 60)^†^	Placebo (*n* = 67)	RLZ 7 mg/kg (*n* = 66)	RLZ 10 mg/kg (*n* = 67)
Age at initial diagnosis, years, mean (SD)	37.1 (10.0)	37.2 (13.7)	43.6 (16.9)	41.8 (20.0)	47.7 (16.0)	42.6 (19.5)	41.4 (19.1)	46.6 (16.0)	42.6 (19.1)
Sex, female, *n* (%)	7 (87.5)	3 (60.0)	7 (87.5)	39 (66.1)	35 (58.3)	28 (46.7)	47 (70.1)	39 (59.1)	35 (52.2)
Race, *n* (%)									
White	8 (100.0)	3 (60.0)	5 (62.5)	39 (66.1)	37 (61.7)	45 (75.0)	46 (68.7)	41 (62.1)	49 (73.1)
Asian	0	2 (40.0)	2 (25.0)	5 (8.5)	7 (11.7)	5 (8.3)	5 (7.5)	9 (13.6)	7 (10.4)
Black or African American	0	0	1 (12.5)	1 (1.7)	0	3 (5.0)	1 (1.5)	0	4 (6.0)
Native Hawaiian or other Pacific Islander	0	0	0	1 (1.7)	0	0	1 (1.5)	0	0
Missing^‡^	0	0	0	13 (22.0)	16 (26.7)	7 (11.7)	14 (20.9)	16 (24.2)	7 (10.4)
Duration of disease, years, mean (SD)	10.2 (9.8)	13.9 (7.6)	5.2 (5.0)	9.1 (9.3)	6.3 (6.5)	10.0 (10.3)	9.4 (9.3)	6.9 (6.8)	9.6 (9.9)
MG-ADL score, mean (SD)	8.8 (3.7)	11.0 (3.5)	9.3 (2.7)	8.4 (3.4)	8.0 (3.6)	8.0 (2.9)	8.4 (3.4)	8.4 (3.8)	8.1 (2.9)
QMG score, mean (SD)	17.9 (4.0)	17.0 (5.8)	14.0 (3.6)	15.6 (3.4)	15.3 (3.6)	15.8 (3.6)	15.8 (3.5)	15.4 (3.7)	15.6 (3.7)
MGFA Disease Class at baseline, *n* (%)									
Class II	1 (12.5)	3 (60.0)	3 (37.5)	22 (37.3)	26 (43.3)	23 (38.3)	23 (34.3)	29 (43.9)	26 (38.8)
Class III	4 (50.0)	2 (40.0)	5 (62.5)	37 (62.7)	31 (51.7)	35 (58.3)	41 (61.2)	34 (51.5)	39 (58.2)
Class IV	3 (37.5)^§^	0	0	0	3 (5.0)	2 (3.3)	3 (4.5)	3 (4.5)	2 (3.0)
Prior MG crisis, *n* (%)	5 (62.5)	3 (60.0)	4 (50.0)	18 (30.5)	16 (26.7)	14 (23.3)	23 (34.3)	19 (28.8)	17 (25.4)
Medications, *n* (%)									
Corticosteroids	5 (62.5)	4 (80.0)	7 (87.5)	34 (57.6)	37 (61.7)	42 (70.0)	38 (56.7)	43 (65.2)	48 (71.6)
Immunosuppressants	2 (25.0)	2 (40.0)	3 (37.5)	31 (52.5)	29 (48.3)	34 (56.7)	33 (49.3)	32 (48.5)	38 (56.7)
Parasympathomimetics^‖^	6 (75.0)	2 (40.0)	5 (62.5)	53 (89.8)	52 (86.7)	53 (88.3)	60 (89.6)	55 (83.3)	57 (85.1)
Thymectomy at baseline, *n* (%)	3 (37.5)	1 (20.0)	0	28 (47.5)	30 (50.0)	20 (33.3)	31 (46.3)	32 (48.5)	20 (29.9)
Total IgG, g/L, mean (SD)	9.5 (3.0)	9.2 (1.0)	9.3 (2.2)	10.3 (2.6)	10.2 (3.3)	9.7 (2.7)	10.2 (2.6)	10.2 (3.2)	9.7 (2.6)

The randomised set consisted of all patients who were randomised, using the treatment assigned instead of the actual treatment received.

* MuSK and AChR autoantibody status were determined based on medical history.

† Includes one patient who had a documented history of both MuSK and AChR autoantibodies.

‡ Data on race were not permitted to be collected in certain countries.

§ Only one patient, who was randomised to the placebo group and had historic MuSK Ab+ gMG, had class IVb disease.

‖ Includes cholinesterase inhibitors.

AChR, acetylcholine receptor; AChR Ab+, acetylcholine receptor autoantibody positive; gMG, generalised myasthenia gravis; IgG, immunoglobulin G; MG, myasthenia gravis; MG-ADL, Myasthenia Gravis Activities of Daily Living; MGFA, Myasthenia Gravis Foundation of America; MuSK, muscle-specific tyrosine kinase; MuSK Ab+, muscle-specific tyrosine kinase autoantibody positive; QMG, Quantitative Myasthenia Gravis; RLZ, rozanolixizumab; SD, standard deviation.

### Efficacy

**Figure 2.**
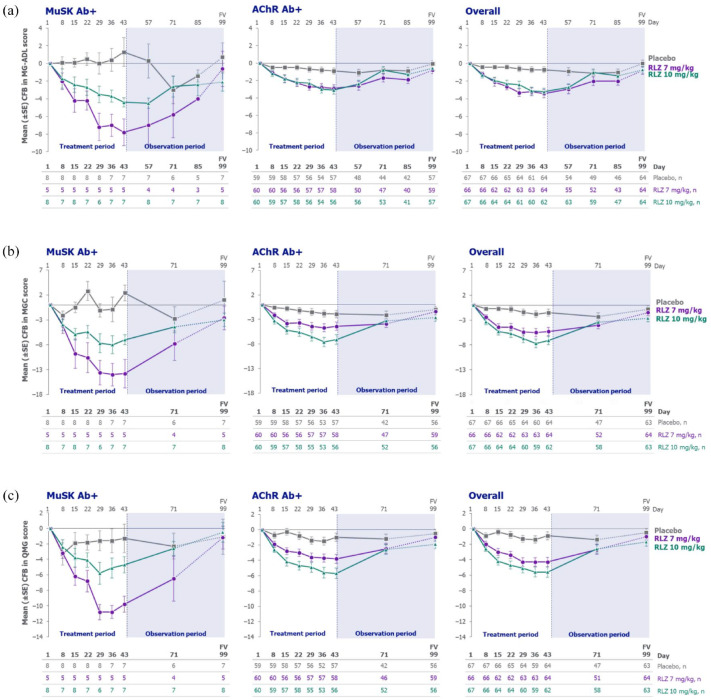
Mean change from baseline in (a) MG-ADL, (b) MGC and (c) QMG scores for patients with MuSK Ab+ gMG, AChR Ab+ gMG and the overall population (randomised set). The figures presenting overall data are reprinted from reference Bril V, et al.^15^ Copyright (2023), with permission from Elsevier. Final visit could occur on any day up to Day 99. AChR Ab+, acetylcholine receptor autoantibody positive; CFB, change from baseline; FV, final visit; gMG, generalised myasthenia gravis; MG-ADL, Myasthenia Gravis Activities of Daily Living; MGC, Myasthenia Gravis Composite; MuSK Ab+, muscle-specific tyrosine kinase autoantibody positive; QMG, Quantitative Myasthenia Gravis; RLZ, rozanolixizumab; SE, standard error.

In patients with MuSK Ab+ gMG who received rozanolixizumab, reductions from baseline to Day 43 in MG-ADL scores were more pronounced than in the overall population (Table 2 and Figure 2(a)). In the overall population at Day 43, both rozanolixizumab dose groups achieved a clinically meaningful and statistically significant least squares mean (LSM) difference from placebo for the change from baseline in MG-ADL score (primary efficacy endpoint).^15^ The rozanolixizumab LSM differences from placebo in MG-ADL scores were numerically greater among patients with MuSK Ab+ gMG compared with the overall population (Table 2).

**Table 2.** Change from baseline at Day 43 in MG-ADL, MGC, QMG and MG Symptoms PRO scale scores in patients with MuSK Ab+ gMG, AChR Ab+ gMG and in the overall population (randomised set).

	MuSK Ab+ population*					AChR Ab+ population*					Overall population				
	Placebo (*n* = 8)^†^	RLZ 7 mg/kg (*n* = 5)		RLZ 10 mg/kg (*n* = 8)^†^		Placebo (*n* = 59)^†^	RLZ 7 mg/kg (*n* = 60)		RLZ 10 mg/kg (*n* = 60)^†^		Placebo (*n* = 67)	RLZ 7 mg/kg (*n* = 66)		RLZ 10 mg/kg (*n* = 67)	
Measure	LSM (SE) CFB	LSM (SE) CFB	LSM difference vs placebo (97.5% CI)	LSM (SE) CFB	LSM difference vs placebo (97.5% CI)	LSM (SE) CFB	LSM (SE) CFB	LSM difference vs placebo (97.5% CI)	LSM (SE) CFB	LSM difference vs placebo (97.5% CI)	LSM (SE) CFB	LSM (SE) CFB	LSM difference vs placebo (97.5% CI)	LSM (SE) CFB	LSM difference vs placebo (97.5% CI)
MG-ADL	2.28 (1.95)	−7.28 (1.94)	−9.56 (−15.25, −3.87)	−4.16 (1.78)	−6.45 (−11.03, −1.86)	−1.10 (0.87)	−3.03 (0.89)	−1.94 (−3.06, −0.81)	−3.36 (0.87)	−2.26 (−3.39, −1.13)	−0.78 (0.49)	−3.37 (0.49)	−2.59 (−3.82, −1.35) *p* < 0.001	−3.40 (0.49)	−2.62 (−3.84, −1.40) *p* < 0.001
MGC	1.40 (2.34)	−14.14 (2.55)	−15.54 (−22.48, −8.59)	−8.56 (2.28)	−9.96 (−15.96, −3.96)	−1.83 (1.64)	−4.45 (1.66)	−2.62 (−4.79, −0.45)	−6.70 (1.64)	−4.88 (−7.06, −2.70)	−2.03 (0.92)	−5.93 (0.92)	−3.90 (−6.32, −1.49) *p* < 0.001	−7.55 (0.93)	−5.53 (−7.93, −3.12) *p* < 0.001
QMG	−3.87 (2.21)	−10.79 (2.51)	−6.92 (−14.24, 0.41)	−7.01 (2.18)	−3.14 (−9.73, 3.45)	−3.09 (1.25)	−6.14 (1.27)	−3.05 (−4.65, −1.44)	−7.77 (1.25)	−4.68 (−6.29, −3.07)	−1.92 (0.68)	−5.40 (0.68)	−3.48 (−5.27, −1.70) *p* < 0.001	−6.67 (0.69)	−4.76 (−6.54, −2.98) *p* < 0.001
MGSPRO Muscle Weakness Fatigability	1.58 (8.93)	−52.61 (10.61)	−54.19 (−85.44, −22.95)	−27.71 (8.70)	−29.29 (−56.61, −1.98)	−10.32 (5.63)	−18.74 (5.72)	−8.42 (−15.46, −1.38)	−23.35 (5.64)	−13.03 (−20.09, −5.96)	−10.59 (3.03)	−23.03 (3.03)	−12.44 (−20.20, −4.68) *p* < 0.001	−25.75 (3.10)	−15.16 (−22.88, −7.44) *p* < 0.001
MGSPRO Physical Fatigue	−4.81 (12.24)	−32.36 (12.16)	−27.55 (−62.45, 7.35)	−30.12 (11.89)	−25.31 (−53.23, 2.61)	−9.23 (5.64)	−14.47 (5.73)	−5.24 (−12.37, 1.89)	−21.78 (5.65)	−12.55 (−19.71, −5.40)	−10.64 (3.05)	−19.29 (3.05)	−8.65 (−16.49, −0.81) *p* = 0.014	−25.46 (3.11)	−14.82 (−22.63, −7.02) *p* < 0.001
MGSPRO Bulbar Muscle Weakness	−12.61 (11.13)	−49.34 (11.97)	−36.73 (−70.74, –‍2.72)	−25.49 (10.54)	−12.88 (−43.03, 17.28)	−6.99 (4.30)	−16.30 (4.37)	−9.31 (−14.88, –3.73)	−17.23 (4.32)	−10.24 (−15.84, −4.63)	−3.52 (2.40)	−14.84 (2.41)	−11.32 (−17.45, −5.20) *p* < 0.001	−14.22 (2.46)	−10.71 (−16.81, −4.60) *p* < 0.001

* All subgroup analyses were descriptive.

† Includes one patient who had a documented history of both MuSK and AChR autoantibodies.

AChR, acetylcholine receptor; AChR Ab+, acetylcholine receptor autoantibody positive; CFB, change from baseline; CI, confidence interval; gMG, generalised myasthenia gravis; LSM, least squares mean; MG-ADL, Myasthenia Gravis Activities of Daily Living; MGC, Myasthenia Gravis Composite; MGSPRO, Myasthenia Gravis Symptoms Patient-Reported Outcome; MuSK, muscle-specific tyrosine kinase; MuSK Ab+, muscle-specific tyrosine kinase autoantibody positive; QMG, Quantitative Myasthenia Gravis; RLZ, rozanolixizumab; SE, standard error.

Rozanolixizumab-treated patients in the overall population also achieved clinically meaningful and statistically significant LSM differences from placebo for the change from baseline to Day 43 in MGC and QMG scores. Statistically significant improvements from baseline in MG Symptoms PRO scale scores were also observed with rozanolixizumab versus placebo (Table 2).^15^ Compared with the overall population, rozanolixizumab-treated patients with MuSK Ab+ gMG showed numerically greater LSM differences from placebo in MGC, QMG and MG Symptoms PRO Muscle Weakness Fatigability, Physical Fatigue and Bulbar Muscle Weakness scores, with the exception of LSM difference from placebo in QMG score for the rozanolixizumab 10 mg/kg group (Table 2).

In patients with MuSK Ab+ gMG, response to rozanolixizumab was rapid, with separation from placebo in MG-ADL and MGC scores observed as early as Day 8 (one week after the first infusion and the first timepoint at which efficacy was assessed; Figure 2(a) and (b)). For QMG scores, separation from placebo was seen by Day 15 in rozanolixizumab-treated patients with MuSK Ab+ gMG (Figure 2(c)). As in the overall population, these improvements in the MuSK Ab+ population were sustained through Day 43, gradually returning towards baseline values during the observation period. Improvements from baseline to Day 43 in MG-ADL, MGC and QMG scores in patients with AChR Ab+ gMG were also consistent with those observed in the overall population (Figure 2). Changes from baseline in MG-ADL, MGC and QMG scores at the individual patient level for patients with MuSK Ab+ or AChR Ab+ gMG are presented in Supplemental Figure 1.

Of the 21 patients with MuSK Ab+ gMG, 19 patients had response data available at Day 43 (rozanolixizumab 7 mg/kg: *n* = 5; rozanolixizumab 10 mg/kg: *n* = 7; placebo: *n* = 7). All 12 (100.0%) rozanolixizumab-treated patients with MuSK Ab+ gMG were MG-ADL and MGC responders (⩾2.0-‍point and ⩾3.0-point improvement, respectively), and 11 (91.7%) were QMG responders (⩾3.0-‍point improvement). In the seven placebo-treated patients with MuSK Ab+ gMG who had available data, responder rates ranged from 0 (MGC score) to 28.6% (*n* = 2; QMG score) (Table 3). The proportion of patients with AChR Ab+ gMG achieving MG-ADL, MGC and QMG response at Day 43 is also reported in Table 3.

**Table 3.** MG-ADL, MGC and QMG responders at Day 43 in patients with MuSK Ab+ gMG, AChR Ab+ gMG and the overall population (randomised set).

	MuSK Ab+ population			AChR Ab+ population			Overall population		
Measure	Placebo (*n* = 7)	RLZ 7 mg/kg (*n* = 5)	RLZ 10 mg/kg (*n* = 7)	Placebo (*n* = 57)	RLZ 7 mg/kg (*n* = 58)	RLZ 10 mg/kg (*n* = 56)	Placebo (*n* = 64)	RLZ 7 mg/kg (*n* = 64)	RLZ 10 mg/kg (*n* = 62)
Responders, *n* (%)									
MG-ADL	1 (14.3)	5 (100)	7 (100)	19 (33.3)	40 (69.0)	37 (66.1)	20 (31.3)	46 (71.9)	43 (69.4)
MGC	0	5 (100)	7 (100)	25 (43.9)	33 (56.9)	40 (71.4)	26 (40.6)	39 (60.9)	46 (74.2)
QMG	2 (28.6)	5 (100)	6 (85.7)	23 (40.4)	30 (51.7)	40 (71.4)	25 (39.1)	35 (54.7)	45 (72.6)

Observed values. Percentages are based on the number of patients with non-missing data on Day 43. MG-ADL responders are defined as having a ⩾2.0-point improvement from baseline; MGC and QMG responders are defined as having a ⩾3.0-point improvement from baseline.

AChR Ab+, acetylcholine receptor autoantibody positive; gMG, generalised myasthenia gravis; MG-ADL, Myasthenia Gravis Activities of Daily Living; MGC, Myasthenia Gravis Composite; MuSK Ab+, muscle-specific tyrosine kinase autoantibody positive; QMG, Quantitative Myasthenia Gravis; RLZ, rozanolixizumab.

In the MuSK Ab+ subgroup, more patients achieved MSE in both rozanolixizumab groups (two [40.0%] patients in the rozanolixizumab 7 mg/kg group and two [25.0%] patients in the rozanolixizumab 10 mg/kg group) than in the placebo group (zero patients). This was consistent with achievement of MSE in the overall population.^15^

### Safety

The proportions of rozanolixizumab-treated patients experiencing any TEAEs and TEAEs considered treatment-related were similar between patients with MuSK Ab+ gMG and the overall population (Table 4). Patients with MuSK Ab+ gMG experienced no severe or serious TEAEs. The most frequently reported TEAEs among patients with MuSK Ab+ gMG were headache, diarrhoea and nausea, while for the overall population, they were headache, diarrhoea and pyrexia. One (12.5%) patient with MuSK Ab+ gMG in the rozanolixizumab 10 mg/kg group permanently discontinued treatment due to TEAEs of epigastric pain and vomiting. No deaths were reported. Full safety data for the overall study population have been reported previously.^15^ The safety profile of rozanolixizumab in patients with AChR Ab+ gMG was similar to that observed in the overall population (Table 4).

**Table 4.** TEAEs in patients with MuSK Ab+ gMG, AChR Ab+ gMG and the overall population (safety set).

	MuSK Ab+ population			AChR Ab+ population			Overall population		
Category	Placebo (*n* = 8)	RLZ 7 mg/kg (*n* = 5)	RLZ 10 mg/kg (*n* = 8)	Placebo (*n* = 59)	RLZ 7 mg/kg (*n* = 58)	RLZ 10 mg/kg (*n* = 62)	Placebo (*n* = 67)	RLZ 7 mg/kg (*n* = 64)*	RLZ 10 mg/kg (*n* = 69)*
Any TEAE, *n* (%)†	3 (37.5)	4 (80.0)	5 (62.5)	41 (69.5)	47 (81.0)	52 (83.9)	45 (67.2)	52 (81.3)	57 (82.6)
Headache	0	2 (40.0)	3 (37.5)	13 (22.0)	27 (46.6)	23 (37.1)	13 (19.4)	29 (45.3)	26 (37.7)
Diarrhoea	0	2 (40.0)	1 (12.5)	9 (15.3)	13 (22.4)	10 (16.1)	9 (13.4)	16 (25.0)	11 (15.9)
Pyrexia	0	0	1 (12.5)	1 (1.7)	8 (13.8)	13 (21.0)	1 (1.5)	8 (12.5)	14 (20.3)
Nausea	0	1 (20.0)	2 (25.0)	5 (8.5)	4 (6.9)	6 (9.7)	5 (7.5)	5 (7.8)	8 (11.6)
Arthralgia	0	0	0	2 (3.4)	3 (5.2)	5 (8.1)	2 (3.0)	4 (6.3)	5 (7.2)
Nasopharyngitis	0	0	1 (12.5)	3 (5.1)	1 (1.7)	4 (6.5)	3 (4.5)	1 (1.6)	5 (7.2)
Urinary tract infection	0	0	1 (12.5)	4 (6.8)	2 (3.4)	1 (1.6)	4 (6.0)	2 (3.1)	2 (2.9)
Myalgia	0	0	0	1 (1.7)	1 (1.7)	4 (6.5)	1 (1.5)	2 (3.1)	4 (5.8)
Vomiting	0	0	1 (12.5)	1 (1.7)	2 (3.4)	3 (4.8)	1 (1.5)	2 (3.1)	4 (5.8)
Hypertension	0	0	0	0	5 (8.6)	0	0	5 (7.8)	0
Any serious TEAE, *n* (%)^‡^	0	0	0	6 (10.2)	5 (8.6)	7 (11.3)	6 (9.0)	5 (7.8)	7 (10.1)
Myasthenia gravis	0	0	0	1 (1.7)	1 (1.7)	2 (3.2)	1 (1.5)	1 (1.6)	2 (2.9)
Myasthenia gravis crisis	0	0	0	2 (3.4)	0	0	2 (3.0)	0	0
Permanent discontinuation from study due to TEAE, *n* (%)	0	0	1 (12.5)	2 (3.4)	2 (3.4)	4 (6.5)	2 (3.0)	2 (3.1)	5 (7.2)
Treatment discontinuation due to TEAE, *n* (%)	0	0	1 (12.5)	2 (3.4)	2 (3.4)	3 (4.8)	2 (3.0)	2 (3.1)	4 (5.8)
Treatment-related TEAEs, *n* (%)	1 (12.5)	2 (40.0)	4 (50.0)	21 (35.6)	30 (51.7)	35 (56.5)	22 (32.8)	32 (50.0)	39 (56.5)
Severe TEAEs, *n* (%)	0	0	0	3 (5.1)	3 (5.2)	13 (21.0)	3 (4.5)	3 (4.7)	13 (18.8)
Deaths, *n* (%)	0	0	0	0	0	0	0	0	0

The safety set consisted of all randomised patients who received at least one dose of rozanolixizumab, analysed according to the actual treatment received. *Two patients in the 7 mg/kg group who incorrectly received 10 mg/kg were analysed in the 10 mg/kg group for safety and PK/PD analyses.

† Specific TEAEs listed are those occurring in ⩾5% of patients in any treatment group in the overall population.

‡ Specific serious TEAEs listed are those occurring in more than one patient in any treatment group in the overall population.

AChR Ab+, acetylcholine receptor autoantibody positive; gMG, generalised myasthenia gravis; MuSK Ab+, muscle-specific tyrosine kinase autoantibody positive; PD, pharmacodynamic; PK, pharmacokinetic; RLZ, rozanolixizumab; TEAE, treatment-emergent adverse event.

### Pharmacodynamics

Within the overall population, reductions in total IgG were observed in the rozanolixizumab groups from the first post-baseline measurement, Day 8, with levels gradually returning to baseline by the end of the observation period.^15^ Observed mean (standard deviation [SD]) maximum percentage change in total IgG from baseline was −71.1% (16.0%) and −77.7% (8.5%) for the rozanolixizumab 7 mg/kg and 10 mg/kg groups, respectively, and −10.6% (9.6%) for the placebo group. Consistent IgG lowering was demonstrated for patients with MuSK Ab+ gMG (mean [SD] maximum percentage change from baseline was −81.6% [10.0%] and −77.0% [5.8%] for the rozanolixizumab 7 mg/kg and 10 mg/kg groups, respectively, and −8.5% [10.1%] for the placebo group).

**Figure 3.**
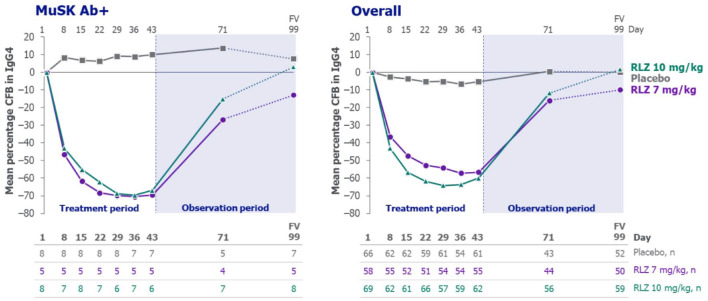
Mean percentage change from baseline in IgG4 concentration in patients with MuSK Ab+ gMG and the overall population (safety set). CFB, change from baseline; FV, final visit; gMG, generalised myasthenia gravis; IgG4, immunoglobulin G4; MuSK Ab+, muscle-specific tyrosine kinase autoantibody positive; RLZ, rozanolixizumab.

For the rozanolixizumab group in the overall and MuSK Ab+ populations, reductions in serum levels of IgG4 were observed as early as Day 8, before returning towards baseline levels during the observation period (Figure 3). The mean (SD) maximum percentage change from baseline in IgG4 in the rozanolixizumab 7 mg/kg, rozanolixizumab 10 mg/kg and placebo groups was –60.7% (23.5%), −67.6% (15.9%) and −14.5% (19.8%), respectively, for the overall population, and −70.5% (4.7%), −68.2% (8.0%) and −0.41% (4.6%), respectively, for patients with MuSK Ab+ gMG. Similarly, reductions from baseline were seen in all other IgG subclasses monitored (IgG1, IgG2 and IgG3; Supplemental Figure 2) for rozanolixizumab-treated patients in both the overall and MuSK Ab+ populations.

## Discussion

The MycarinG study is the largest phase III clinical study of patients with gMG to date. The proportion of patients with MuSK Ab+ gMG enrolled in the study (10.5% [*n* = 21]) was representative of the proportion of patients with this subtype of MG in the real world (5%–8% of patients).^7^ Further, the number of patients with MuSK Ab+ gMG is the largest to be enrolled in a randomised controlled phase III study. In this subgroup analysis, treatment with rozanolixizumab demonstrated improvements from baseline in multiple MG-specific endpoints in patients with MuSK Ab+ gMG, consistent with those observed in the overall population. Both rozanolixizumab doses were well tolerated.

Numerically greater improvements from baseline in MG-ADL, MGC, QMG and MG Symptoms PRO scale scores at Day 43 were observed with rozanolixizumab 7 mg/kg compared with rozanolixizumab 10 mg/kg in patients with MuSK Ab+ gMG. Conversely, in the overall population, improvements in several efficacy endpoints appeared to be greater with rozanolixizumab 10 mg/kg than with 7 mg/kg.^15^ This may be explained by the imbalance in baseline characteristics within the MuSK Ab+ gMG subgroup; patients treated with rozanolixizumab 7 mg/kg had higher MG-ADL, MGC, QMG and MG Symptoms PRO scale scores at baseline (data not presented for MGC and MG Symptoms PRO scale baseline scores) compared with those receiving rozanolixizumab 10 mg/kg, allowing more scope for improvement in scores. However, due to the small number of patients with MuSK Ab+ gMG in each dosing group, the apparent larger effect of the lower dose in MuSK patients may simply be due to chance.

Rozanolixizumab treatment improved physical fatigue in patients with MuSK Ab+ gMG as measured by a reduction from baseline to Day 43 in MG Symptoms PRO Physical Fatigue scores. Physical fatigue has been identified as an important symptom for patients with MG.^28^ In the MycarinG study, use of the MG Symptoms PRO measure facilitated comprehensive assessment of physical fatigue and its manifestations, such as lack of energy, muscle weakness and heaviness in the body and limbs.^29^ Hence, inclusion of the MG Symptoms PRO complemented other MG-specific patient- and clinician-reported outcome measures used in the study that do not fully capture physical fatigue.^24,29^

Patients were required to have a previously documented positive record of autoantibodies against MuSK or AChR at screening. Six patients with a documented history of MuSK Ab+ gMG tested negative for the presence of MuSK autoantibodies at baseline. Autoantibodies of low abundance may not be detectable by classical assays, and it is known that immunosuppressive treatment influences autoantibody titres. Changes in disease severity have also been shown to correlate with changes in autoantibody titres in individual patients.^4,30–32^ Autoantibody levels may also be impacted by other treatments, for example those that suppress B-cell proliferation.^33^ However, patients who had received treatment with rituximab 6 months prior to baseline, or 12 months prior if B cells had not returned to the normal range, were excluded from the MycarinG study. Further, no patterns in prior treatment with IVIg or plasma exchange were observed that could be deemed responsible for the changes in autoantibody status. Patients who subsequently test negative for MG-specific autoantibodies may still be considered positive for autoantibodies against MuSK or AChR, and show a response to immunotherapy.^34^ This may explain why all five of the patients in this group who received rozanolixizumab were MG-ADL responders.

Patients with MuSK or AChR Ab+ gMG have shown high rates of response to plasma exchange;^35^ therefore, it can be reasonably assumed that they are both responsive to other IgG-lowering therapies. Rozanolixizumab led to rapid reductions in total IgG concentrations, with a robust lowering of IgG4 observed in patients with MuSK Ab+ gMG. This was in line with total IgG lowering observed following rozanolixizumab treatment in the overall population.

A limitation of this analysis was the small patient numbers within the MuSK Ab+ gMG treatment groups, which may limit generalisability of the results to the wider MuSK Ab+ gMG population. While the proportion of patients with MuSK Ab+ gMG in the study was reflective of the lower prevalence of MuSK Ab+ gMG in the real-world gMG population, the small patient numbers led to imbalances in the baseline disease characteristics between treatment groups. Despite this, the observed baseline demographics and characteristics were broadly reflective of those described in the literature for patients with MuSK Ab+ gMG.^5,36^ For example, a high proportion of patients with MuSK Ab+ gMG in the study were female and had prior MG crisis, and as expected, a low proportion had prior thymectomy.

Due to nuances in the clinical characteristics and poor response to standard treatments considered for gMG, MuSK Ab+ gMG presents a high burden of disease for patients.^5,7,9^ The disease phenotype is often associated with greater bulbar symptom severity and more frequent myasthenic crises than AChR Ab+ gMG, and standard therapies for gMG are not always effective.^5,7^ Therefore, there is an urgent need for effective targeted treatments in this patient population.

Primary results reported for the MycarinG study provided support for FcRn inhibition in gMG,^15^ and when combined with the results of this subgroup analysis, provide evidence for rozanolixizumab treatment in patients with MuSK Ab+ gMG. However, longer follow-up of these patients is required to demonstrate continuous benefits of rozanolixizumab treatment in the MuSK Ab+ gMG population. The safety and efficacy of repeated 6-week treatment cycles was investigated in the OLE study, MG0007.^37^

## Conclusion

Owing to the limited treatment options and severe clinical disease subtype, patients with MuSK Ab+ gMG are a population with a high unmet need. The subgroup of patients with MuSK Ab+ gMG enrolled in the MycarinG study is the largest population with MuSK Ab+ gMG included in any randomised controlled phase III study to date. Across all endpoints, patients with MuSK Ab+ gMG showed a rapid, robust clinical response to treatment with rozanolixizumab, consistent with that observed for the overall population. The pharmacodynamic data, which showed lowering of IgG4 in line with total IgG, further support the efficacy of rozanolixizumab in these patients. As observed in the overall population, both doses of rozanolixizumab were well tolerated. Together, these data support the use of rozanolixizumab as a treatment option for patients with MuSK Ab+ gMG.

## Supplemental Material

sj-docx-1-tan-10.1177_17562864241273036 – Supplemental material for Efficacy and safety of rozanolixizumab in patients with muscle-specific tyrosine kinase autoantibody-positive generalised myasthenia gravis: a subgroup analysis of the randomised, double-blind, placebo-controlled, adaptive phase III MycarinG study Supplemental material, sj-docx-1-tan-10.1177_17562864241273036 for Efficacy and safety of rozanolixizumab in patients with muscle-specific tyrosine kinase autoantibody-positive generalised myasthenia gravis: a subgroup analysis of the randomised, double-blind, placebo-controlled, adaptive phase III MycarinG study by Ali A. Habib, Sabrina Sacconi, Giovanni Antonini, Elena Cortés-Vicente, Julian Grosskreutz, Zabeen Mahuwala, Renato Mantegazza, Robert M. Pascuzzi, Kimiaki Utsugisawa, John Vissing, Tuan Vu, Heinz Wiendl, Marion Boehnlein, Bernhard Greve, Franz Woltering and Vera Bril in Therapeutic Advances in Neurological Disorders

## Declarations

**Ethics approval and consent to participate:** The study was carried out in compliance with the International Conference on Harmonisation Good Clinical Practice guidelines and the principles of the Declaration of Helsinki. The study protocol, amendments and patients’ informed consent were reviewed by a national, regional or independent ethics committee or institutional review board for each participating site. The approving ethics committees or institutional review boards are listed in the Supplemental Material. All patients provided written informed consent.

**Consent for publication:** Not applicable.

**Competing interests:** The authors declared the following potential conflicts of interest with respect to the research, authorship, and/or publication of this article: AAH has received research support from Alexion Pharmaceuticals, argenx, Cabaletta Bio, Genentech/Roche, Immunovant, Regeneron Pharmaceuticals, UCB Pharma and Viela Bio (now Amgen). He has received honoraria from Alexion Pharmaceuticals, Alpine Immune Sciences, argenx, Genentech/Roche, Immunovant, Inhibrx, Regeneron Pharmaceuticals, NMD Pharma and UCB Pharma. SS has nothing to disclose. GA has received funding for travel, conference and meeting attendance or advisory board participation from Alexion Pharmaceuticals, argenx, Janssen Pharmaceuticals (now Johnson & Johnson Innovative Medicine), Kedrion and UCB Pharma. EC-V receives public speaking honoraria and compensation for advisory boards and/or consultation fees from Alexion Pharmaceuticals, argenx, Janssen Pharmaceuticals (now Johnson & Johnson Innovative Medicine) and UCB Pharma. JG has served as a Consultant for Alexion Pharmaceuticals, Biogen and UCB Pharma, and his institution has received research support from the Boris Canessa Foundation. ZKM has received funding for advisory board participation from Alexion Pharmaceuticals. RM has received funding for travel and meeting attendance or advisory board participation from Alexion Pharmaceuticals, argenx, BioMarin, Catalyst, Sanofi, Regeneron Pharmaceuticals and UCB Pharma. RMP has nothing to disclose. KU has served as a paid Consultant for argenx, Chugai Pharmaceutical, HanAll Biopharma, Janssen Pharmaceuticals (now Johnson & Johnson Innovative Medicine), Merck, Mitsubishi Tanabe Pharma, UCB Pharma and Viela Bio (now Amgen); he has received speaker honoraria from Alexion Pharmaceuticals, argenx, the Japan Blood Products Organization and UCB Pharma. JV has been a Consultant on advisory boards for Amicus Therapeutics, Arvinas, Biogen, Fulcrum Therapeutics, Genethon, Horizon Therapeutics (now Amgen), Lupin, ML Biopharma, Novartis, Regeneron Pharmaceuticals, Roche, Sanofi Genzyme (now Sanofi), Sarepta Therapeutics and UCB Pharma. He has received research, travel support and/or speaker honoraria from Alexion Pharmaceuticals, argenx, Biogen, Edgewise Therapeutics, Fulcrum Therapeutics, Lupin, Sanofi Genzyme (now Sanofi) and UCB Pharma. He is a Principal Investigator in clinical trials for Alexion Pharmaceuticals, argenx, Genethon, Horizon Therapeutics (now Amgen), Janssen Pharmaceuticals (now Johnson & Johnson Innovative Medicine), ML Biopharma, Novartis, Regeneron Pharmaceuticals, Roche, Sanofi Genzyme (now Sanofi) and UCB Pharma. TV is the USF Site Principal Investigator for MG clinical trials sponsored by Alexion Pharmaceuticals, Amgen, argenx, Cartesian Therapeutics, Dianthus Therapeutics, Immunovant, Johnson & Johnson, Regeneron Pharmaceuticals and UCB Pharma, and has served as a speaker for Alexion Pharmaceuticals, argenx, and CSL Behring. He performed consulting work for Alexion Pharmaceuticals, argenx, Dianthus Therapeutics, ImmunAbs and UCB Pharma. HW is a Scientific Advisor for AbbVie, Alexion Pharmaceuticals, argenx, Bristol Myers Squibb/Celgene, Janssen Pharmaceuticals (now Johnson & Johnson Innovative Medicine), Merck, Novartis and Sandoz. He has received speaker honoraria and travel support from Alexion Pharmaceuticals, Biogen, Bristol Myers Squibb, Genzyme, Merck, Neurodiem, Novartis, Ology, Roche, TEVA and WebMD Global and is a paid Consultant for AbbVie, Actelion, argenx, BD, Biogen, Bristol Myers Squibb, EMD Serono, Fondazione Cariplo, Gossamer Bio, Idorsia, Immunic, Immunovant, Janssen Pharmaceuticals (now Johnson & Johnson Innovative Medicine), Lundbeck, Merck, NexGen, Novartis, PSI CRO, Roche, Sanofi, the Swiss Multiple Sclerosis Society, UCB Pharma and Worldwide Clinical Trials. His research is funded by the German Ministry for Education and Research, Deutsche Forschungsgesellschaft, Deutsche Myasthenie Gesellschaft e.V., Alexion Pharmaceuticals, Amicus Therapeutics Inc., argenx, Biogen, CSL Behring, F. Hoffmann-La Roche, Genzyme, Merck KgaA, Novartis, Roche and UCB Pharma. MB, BG and FW are employees and shareholders of UCB Pharma. VB is a Consultant for Akcea, Alexion Pharmaceuticals, Alnylam, argenx, CSL, Grifols, Ionis, Immunovant, Janssen Pharmaceuticals (now Johnson & Johnson Innovative Medicine), Momenta (now Johnson and Johnson), Novo Nordisk, Octapharma, Pfizer, Powell Mansfield, Roche, Sanofi, Takeda and UCB Pharma. She has received research support from Akcea, Alexion Pharmaceuticals, argenx, CSL, Grifols, Immunovant, Ionis, Momenta (now Johnson and Johnson), Octapharma, Takeda Pharmaceuticals, UCB Pharma and Viela Bio (now Amgen).

**Availability of data and materials:** Underlying data from this manuscript may be requested by qualified researchers 6 months after product approval in the US and/or Europe, or global development is discontinued, and 18 months after trial completion. Investigators may request access to anonymised individual patient-level data and redacted trial documents which may include: analysis-ready datasets, study protocol, annotated case report forms, statistical analysis plans, dataset specifications, and clinical study reports. Prior to use of the data, proposals need to be approved by an independent review panel at www.Vivli.org and a signed data-sharing agreement will need to be executed. All documents are available in English only, for a pre-specified time, typically 12 months, on a password-protected portal.
